# Case Report: β-thalassemia major on the East African coast

**DOI:** 10.12688/wellcomeopenres.17907.1

**Published:** 2022-07-13

**Authors:** Alexander W. Macharia, George Mochamah, Johnstone Makale, Thad Howard, Neema Mturi, Peter Olupot-Olupot, Anna Färnert, Russell E. Ware, Thomas N. Williams

**Affiliations:** 1 Epidemiology and Demography Department, KEMRI/Wellcome Trust Kilifi, Kilifi, 254, Kenya; 2 Division of Hematology, Cincinnati Children's Hospital Medical Center, OH, USA; 3 Mbale Clinical Research Institute, Mbale, Uganda; 4 Busitema University Faculty of Health Sciences, Mbale, Uganda; 5 Karolinska Institute, Stockholm, Sweden; 6 Institute for Global Health Innovation, Imperial College, London, UK

**Keywords:** β-thalassemia major, rs33941849, East Africa, HbA2, sequencing

## Abstract

**Background:** β-thalassemia is rare in sub-Saharan Africa and to our knowledge there has been no case of homozygous β-thalassemia major reported from this region. In a recent cohort study, we identified four β-thalassemia mutations among 83 heterozygous carriers in Kilifi, Kenya. One of the mutations identified was a rare β-globin gene initiation codon mutation (ATG➝ACG) (rs33941849). Here we present a patient with β-thalassemia major resulting from this mutation, only the second homozygous patient to have been reported.

**Methods:** The female patient presented to Kilifi County Hospital aged two years with a one week left sided abdominal swelling. Clinical, hematological and genetic information were collected at admission and follow-up.

**Results: **Admission bloods revealed marked anemia, with a hemoglobin (Hb) value of 6.6 g/dL and a low mean corpuscular volume of 64 fL. High performance liquid chromatography (HPLC) revealed the absence of HbA0 and elevated levels of HbF, suggesting a diagnosis of β-thalassemia major. Sequencing revealed that the child was homozygous for the rs33941849 initiation codon mutation.

**Conclusions: **We hope that this study will create awareness regarding the presence of β-thalassemia as a potential public health problem in the East Africa region and will prompt the development of local guidelines regarding the diagnosis and management of this condition.

## Introduction

β-thalassemia is rare in most of Africa, with the exception of North Africa where the prevalence, causal pathogenic variants and disease outcomes have all been well described previously ( Hamamy & Al-Allawi, 2013). We recently reported elevated levels of HbA _2,_ suggestive of β-thalassemia, in a small proportion of children participating in a cohort study conducted in Kilifi county on the coast of Kenya ( Macharia *et al*., 2019). We subsequently sequenced samples from the same children and found that 0.6% were carriers of one of four different β-thalassemia pathogenic variants: the β ^0^-thalassemia variants CD22 (GAA➝TAA) (rs33959855), initiation codon (ATG➝ACG) (rs33941849) and IVS1-3ʹ end del 25bp (rs193922563) and the β ^+^-thalassemia variant IVS-I-110 (G➝A) (rs35004220). Whereas the mutations observed in North Africa resemble those found in Middle Eastern countries, those identified in Kilifi were a mixture of mutations reported from Asia and the Middle East. To the best of our knowledge, no cases of β-thalassemia major – a condition in which both *HBB* genes are affected by a β ^0^-thalassemia mutation to result in the complete loss of normal β ^0^-globin production - have yet been reported from the East Africa region. Here, we describe what is, to the best of our knowledge, the first case of β ^0^-thalassemia major to be recognised from within this region.

### Ethics

Written informed consent was provided by the parents of the study participant. Ethical approval for the study was granted by the Kenya Medical Research Institute Ethical Review Committee in Nairobi, Kenya (Number: SCC3891).

### Patient report

The child, a two-and-a-half-year-old female, presented to Kilifi County Hospital in Kenya, with a one-week history of left sided abdominal swelling. No previous hospital admissions were reported. Clinical history suggested delayed developmental milestones; specifically, she was unable to walk without support. The child was the fourth born of five siblings, all of whom were alive and well as were both of her parents. Both her parents were of Mijikenda ethnolinguistic ancestry and no recent genetic admixture was apparent from the clinical history. On physical examination, the child was pale but had no signs of clinical jaundice. Her vital signs were essentially normal with the exception of a fever measured at 38.8 ^°^C per axilla. Fronto-maxillary skull bossing was apparent. Her abdomen was distended, soft and non-tender, massive splenomegaly being detected at 8cm below the costal margin. She was severely malnourished with a weight of 8.8 kg, a height of 78.5 cm, a height for age z-score (HAZ) of -3.79, a weight for age z-score (WAZ) of -3.20 and a weight for height z-score (WHZ) of -1.23. Further examination was essentially normal. The timeline of events is given in Table 1.

**Table 1.** Timeline of events.

Age at presentation	Symptoms	Diagnostic Testing	Interventions
2 years 6 months	• Delayed developmental milestones • Left sided abdominal swelling • 8 prior transfusions (other hospital) • Low grade fever • Bossing of the skull • Massive splenomegaly • Malnutrition	• HPLC analysis; absence of HbA and elevated HbF (>80% of total Hb) • Sequenced her *HBB* gene region, which revealed she was homozygous for the initiation codon (ATG➝ACG) mutation (rs33941849) • Full haemogram; hb 6.6gm/dl	• Admission • Treated for suspected sepsis
3 years 11 months	• Cough • Fever • Splenomegaly • Malnutrition • Transfusion dependent anaemia	• PCR for the α ^-3.7^ deletional form of α-thalassemia was negative • Repeat HPLC analysis revealed the continued absence of HbA, HbF (>80% of total Hb) and HbA2 at 5%	• Began regular monthly blood transfusions • Referred for surgical splenectomy

A full hemogram revealed marked anemia (Hb 6.6 g/dL), a low mean corpuscular volume (MCV) of 64 fL, a low mean corpuscular hemoglobin (MCH) of 19.4 pg, and a raised total white blood cell (WBC) count of 49.6 × 10 ^9^/µl which were predominantly lymphocytes. Her platelet count was normal at 321 × 10 ^6^/L and her creatinine mildly elevated at 32 μmol/l. Blood cultures and tests for malaria were negative. A peripheral blood film revealed nucleated red blood cells (RBCs), microcytes, dacrocytes, acanthocytes, giant platelets and a marked lymphocytosis ( Table 2).

**Table 2.** Complete blood count and peripheral blood film from the child with β-thalassemia.

Parameter	First admission ^¢^	Second Admission ^‡^
HbA _2_ (%)	2.5	5.0
HbF (%)	>80%	>80%
WBC count (×10 ^3^/μl)	49.6	20.5
RBC count (×10 ^6^/μl)	3.38	1.10
Hb (g/dl)	6.6	2.2
HCT (%)	21.5	6.2
MCV (fL)	64	56
MCH (pg)	19.4	20.1
Platelets (×10 ^6^/L)	321	330
Peripheral Blood Film	Leucocytosis, lymphocytosis, Nucleated red blood cells, dacrocytes, anisocytosis and giant platelets	

Abbreviations: WBC, white blood cells; RBC, red blood cells; Hb, hemoglobin; HCT, hematocrit; MCV, mean cell volume; MCH, mean cell hemoglobin; PBF, peripheral blood film. ¢Age=2.5 years, ‡Age=3.5 years.

The child was admitted to the general pediatric ward with a working diagnosis of iron deficiency anemia, potentially complicated by bacterial sepsis, and with a differential diagnosis of sickle cell anemia. She was treated empirically with iron and folic acid supplementation for her anemia and with intravenous penicillin and gentamicin to cover sepsis. She was also prescribed malaria prophylaxis with proguanil pending analysis for sickle cell anemia by high-performance liquid chromatography (HPLC). Her fever subsided within two days of admission, at which point she was discharged home on oral amoxicillin, with follow-up planned for the following week.

**Figure 1.**
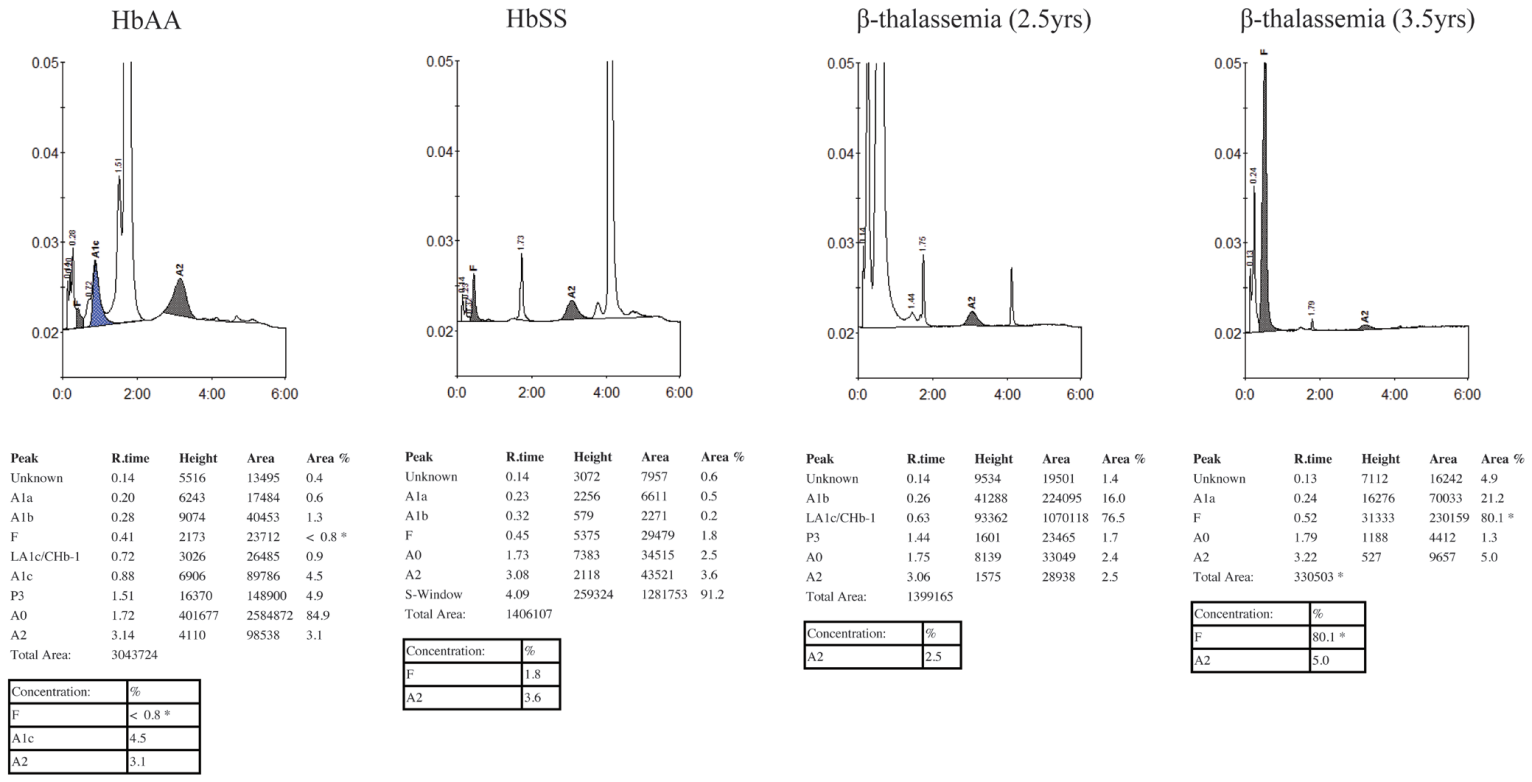
HPLC chromatograms from study participants with normal hemoglobin A individual (HbAA), homozygous hemoglobin S (HbSS) and homozygous β-thalassemia patient at first admission (age 2.5 years) and at second admission (age 3.5 years).

The results of her HPLC analysis, received after discharge from hospital, revealed the absence of normal adult hemoglobin (HbA), normal levels of HbA _2 _at 2.5% and elevated levels of fetal hemoglobin (HbF) (>80% of total Hb) that eluted in adjacent peaks A1b (16%) and LA1C/cHb1 (76.5%) ( Figure 1). The complete absence of HbA suggested a diagnosis of β ^0^-thalassemia major. We therefore sequenced her *HBB* gene region as described in detail previously ( Clark & Thein, 2004), which revealed that the child was homozygous for the initiation codon (ATG➝ACG) mutation (rs33941849).

Initially lost to follow-up, the child re-presented at the age of three years 11 months with a one-week history of a cough and fever. On examination at that time, her spleen remained grossly enlarged at 10 cm, and she remained malnourished with a HAZ of -4.98, a WAZ of -4.01 and a WHZ of -0.99. Although hemodynamically stable, she was profoundly anemic (Hb 2.2 g/dL) and was therefore transfused and treated with folic acid supplementation and nutritional support. Repeat HPLC analysis revealed the continued absence of HbA together with elevated levels of HbF (>80%) and HbA _2 _ (at 5%) ( Figure 1). PCR for the α ^-3.7^ deletional form of α-thalassemia was negative.

## Discussion

To the best of our knowledge, this is the first case of homozygous β-thalassemia to be reported from the East Africa region. The mutation responsible disrupts the transfer RNA binding site to result in a β ^0^ form of the disease. It appears to be rare in other populations: only 45 carriers have been reported in the literature to date, 20 of which were from our recently reported study ( Macharia *et al*., 2020). Other reports of carriers have come from a wide range of countries including Switzerland ( Beris *et al*., 1993), Belgium ( Wildmann *et al*., 1993), Russia ( Molchanova *et al*., 1998), India ( Gorakshakar *et al*., 2018; Gupta *et al*., 2002) and the former Yugoslavia ( Jankovic *et al*., 1990). The only homozygous case described to date was a male child of Pakistani origin who presented at 10 months of age with a palpable liver and spleen at 7 cm and 3 cm below costal margin, respectively. His Hb was 9.2 g/dl, MCV of 73 fl and MCH of 33 pg. He was also found to be homozygous for the α ^-3.7^-thalassemia deletion and to have a Bantu β-globin gene cluster haplotype. He was managed with regular blood transfusions ( Khan *et al*., 2000).

On comparing the current and previously described cases, all had anemia, a low MCV and massive splenomegaly. In our current patient, we also observed elevated levels of HbF and varying levels of HbA _2_ at the two points of testing, an observation which is common in β-thalassemia major ( Steinberg & Rodgers, 2015). Options for the treatment of this condition in our context are limited. Throughout much of the world, first line management includes the provision of regular, leuco-depleted blood transfusions together with extended antigen typing of transfused blood to reduce the risk of alloimmunization. Iron-chelation is also used to mitigate the risk of iron overload ( Steinberg *et al*., 2009) while more recently, allogeneic hematopoietic cell transplantation (HCT) is also being used as a potentially curative therapy. However, all these strategies are beyond the capacity of our local health-care system. Nevertheless, there is growing evidence to support the use of hydroxyurea, an HbF inducer, in the treatment of transfusion and non-transfusion dependent β-thalassemia ( Algiraigri *et al*., 2017a; Algiraigri *et al*., 2017b). We will investigate this strategy together with surgical splenectomy if the child returns for further follow-up in the hope that these will reduce the frequency at which transfusions will be required. 

## Conclusions

We have previously estimated the birth prevalence of β-thalassemia major in our local community at approximately 1 in 100,000 ( Macharia *et al*., 2020). Nevertheless, low awareness of this condition among clinicians and the low availability of diagnostic facilities within the region mean that historically, individuals with β-thalassemia major have probably been misdiagnosed with other conditions such as sickle cell anemia or iron deficiency anemia as was the case with this child. As such, we hope that our case study will raise awareness about the existence and clinical importance of β-thalassemia major as a public health problem within the East Africa region and lead to the development of locally appropriate diagnostic and treatment guidelines.

## Data availability

### Underlying data

All data underlying the results are available as part of the article and no additional source data are required.

## Consent

Written informed consent for publication of their clinical details was obtained from the parents of the patient.
