# Supplementary material for: Case Report: β-thalassemia major on the East African coast
Source: Wellcome Open Res. 2022 Jul 13;7:188. [Version 1] doi: 10.12688/wellcomeopenres.17907.1 (PMC10551670; doi:10.12688/wellcomeopenres.17907.1)
Supplement: Peer review history

# Reviewer report: Reviewer response for version 1

**DOI:** 10.21956/wellcomeopenres.19844.r67871

**Published:** 2023-10-04

**Authors:** Paloma Ropero

**Affiliations:** 1 Hospital Clínico San Carlos, Madrid, Spain

This work presents the case of a baby with homozygous beta-thalassemia in a region where this had not been previously reported.

 The report is interesting because it shows that there could be similar cases and confuse the diagnosis with sickle cell anemia or severe iron deficiency.

 This study provides valuable information about β-thalassemia in a region where it is rare and presents a rare clinical case that may help improve the diagnosis and treatment of this condition in the future.

 Unfortunately, local conditions hinder the ability to provide optimal treatment.

 Do you know if the parents are family? Are they related?

Are enough details provided of any physical examination and diagnostic tests, treatment given and outcomes?

Yes

Is the case presented with sufficient detail to be useful for other practitioners?

Yes

Is sufficient discussion included of the importance of the findings and their relevance to future understanding of disease processes, diagnosis or treatment?

Yes

Is the background of the case’s history and progression described in sufficient detail?

Yes

Reviewer Expertise:

Hemoglobinopathies and thalassemia

I confirm that I have read this submission and believe that I have an appropriate level of expertise to confirm that it is of an acceptable scientific standard.

# Reviewer report: Reviewer response for version 1

**DOI:** 10.21956/wellcomeopenres.19844.r51556

**Published:** 2022-07-25

**Authors:** Martin H. Steinberg

**Affiliations:** 1 Department of Medicine, Boston Medical Center, Boston, MA, USA

This is a convincing report of an infant with homozygous beta-thalassemia from a region where this has not been previously reported. The report is worthwhile since this was, and other similar cases could be mistaken for sickle cell anemia or severe iron deficiency. 

 The marked lymphocytosis seems unexplained, but this does not detract from the main point of the report. Unfortunately, local conditions preclude optimal treatment.

Are enough details provided of any physical examination and diagnostic tests, treatment given and outcomes?

Yes

Is the case presented with sufficient detail to be useful for other practitioners?

Yes

Is sufficient discussion included of the importance of the findings and their relevance to future understanding of disease processes, diagnosis or treatment?

Yes

Is the background of the case’s history and progression described in sufficient detail?

Yes

Reviewer Expertise:

disorders of hemoglobin

I confirm that I have read this submission and believe that I have an appropriate level of expertise to confirm that it is of an acceptable scientific standard.
